# The Relative Age Effect and Talent Identification Factors in Youth Volleyball in Poland

**DOI:** 10.3389/fpsyg.2020.01445

**Published:** 2020-07-07

**Authors:** Krystian Rubajczyk, Andrzej Rokita

**Affiliations:** Department of Team Games Sport, University School of Physical Education in Wrocław, Wrocław, Poland

**Keywords:** selection, youth development, calendar age, maturity, sports success

## Abstract

Previous studies in team sports have not reported evidence regarding the relative age effect (RAE) in relation to the talent identification (TI) process in volleyball, which is organized and controlled by a national federation. Volleyball is a non-contact team sport in which a player’s physique does not directly affect other players in the game but is considered one of the most critical factors in the TI process. The aims of the present study were (1) to determine the differences in the quarterly distribution of age between Polish youth volleyball players from the Olympic Hopes Tournament (OHT) and the general population, (2) to investigate the quarterly differences in anthropometric characteristics and motor test results in OHT participants, and (3) to identify the criteria that determine selection for the National Volleyball Development Program (NVDP). The present study identified the RAE in young male (*n* = 2,528) and female (*n* = 2,441) Polish volleyball players between 14 and 15 years of age who competed in the elite OHT in 2004–2015. The study included anthropometric characteristics, motor test results, and selection for the NVDP. The multivariate analysis of covariance demonstrated no significant main effect for birth quarter or calendar age in any of the OHT female players or in male players selected for the NVDP. In the group of non-selected NVDP male players, the analysis demonstrated significant differences by birth quarter as a covariate for body height (*F* = 0.01, *p* < 0.001), spike reach (*F* = 7.33, *p* < 0.05), and block jump (*F* = 0.02, *p* < 0.001). Significant differences by calendar age as a covariate were observed for body mass (*F* = 0.53, *p* < 0.01), spike jump (*F* = 2.64, *p* < 0.05), block jump (*F* = 0.4, *p* < 0.01), and zigzag agility test results (*F* = 0.01, *p* < 0.01). The results showed a significant overrepresentation of early-born participants in the OHT and NVDP subsamples. The classification model demonstrated that a combination of four characteristics optimally discriminated between players selected for the NVDP and those who were not selected. This combination of variables correctly classified 77.7% of the female players and 71.8% of the male players in terms of their selection for the NVDP. The results of this study show that jumping ability and body height are crucial in the TI and selection process in youth volleyball.

## Introduction

The requirements of youth sports lead to the age banding of players into training groups and teams; sports administrators age-band players into training groups relative to cutoff dates (e.g., the start and end of the calendar year; Cobley et al., 2009). The assessment of players by trainers during the talent identification (TI) process can be disrupted by differences in the players’ biological development (Ramos et al., 2019) and sociological factors (Hancock et al., 2013). Players born closer to the starting point of their age group relative to their peers may be older by as much as 2 to 5 years (Johnson et al., 2017), and the selection of more mature and stronger players will result in an overrepresentation of players born in the first part of the selection period (e.g., quarter). As a consequence, in youth ball sports, later-born and less mature players are strongly underrepresented, especially at the elite level (Hill and Sotiriadou, 2016). This phenomenon is a well-documented selection bias and is known as the relative age effect (RAE; Musch and Grondin, 2001).

The presence of an RAE has been observed at the senior and youth levels in the following contact team sports: basketball (Arrieta et al., 2016; Werneck et al., 2016), soccer (González-Víllora et al., 2015; Skorski et al., 2016), and handball (Schorer et al., 2009). Contrary, the RAE was not found in other team sports such as rugby (Jones et al., 2018) and water polo (Barrenetxea-Garcia et al., 2018). In line with this, the findings of existing literature on RAE in contact team sports have been controversial so far. Nevertheless, it is reported that discrimination against players born in the last quarter of a calendar year differs, depending on the position, gender, age of the player (Salinero et al., 2013; Lidor et al., 2014), and expertise level (Praxedes et al., 2017). Volleyball, however, is a non-contact team sport in which a player’s physique does not directly affect other players in the game. It was reported that more than two-thirds of all points scored in volleyball are due to short dynamic bouts that mainly depend on players’ vertical jump and body height (Silva et al., 2014). Interestingly, only a few works have considered RAE in terms of birth-date discrimination in volleyball. An overrepresentation of players born in the first quarter of the year compared to other quarters was observed in a group of young male and female players and in the players in the age group younger than 19 years to younger than 23 years in men’s World Volleyball Championship (Okazaki et al., 2011; Nakata and Sakamoto, 2012; Campos et al., 2016). In addition, the RAE in volleyball has been identified in school competitions (Reed et al., 2017). Research by Lupo et al. (2019) emphasizes the different nature of the RAE in volleyball compared to other elite team sports in Italy.

Considering the aforementioned, it is clear that RAE manifests itself in such team games according to the physical characteristics of the player. Previous studies about the potential advantage in the physical and motor abilities of early-born players to their counterparts were carried out mostly in the field of other team sports. For example, in youth soccer, possible differences in biological maturation and anaerobic characteristics were observed between players born in the first and fourth quarters of the year (Deprez et al., 2013). Nevertheless, a pilot study from Papadopoulou et al. (2019) shows no quarter differences in anthropometric and physiological characteristics in youth volleyball female players. In contrast, late-born youth basketball players have a “double disadvantage” in body height compared to their peers (Rubajczyk et al., 2017). In addition, advanced maturity status and being relatively older affected players’ game-related specific fitness (Duarte et al., 2019). However, the RAE has not been thoroughly explored in volleyball, especially with regard to the TI process.

The TI process in volleyball may be challenging for practitioners. In general, successful discrimination between talented and untalented-identified junior volleyball players is multidimensional and is based on the assessment of skill attributes, a tactical understanding of the game (Jager and Schollhorn, 2007), or game intelligence (Rikberg and Raudsepp, 2011), perceptual-cognitive skills (Alves et al., 2013), motor abilities, and anthropometric and physical characteristics (Marcelino et al., 2014). Despite this, body height is considered a key criterion in the TI process used to assess youth players (Aouadi et al., 2012; Carvalho et al., 2020). Thus, the failure to estimate the adult body height of an athlete will significantly hinder the effective TI process in volleyball (Baxter-Jones et al., 2020). In addition, maturity-associated variation in performance (Sandercock et al., 2013), and sex differences in the onset of puberty (Malina, 2014; Kwieciński et al., 2018) may indicate an ineffective TI process and maintain the existence of the RAE phenomenon in youth sports. Furthermore, in a non-contact team sport such as volleyball, earlier age at the start of peak height velocity and player body height may not be important performance factors but can be decisive factors in TI.

An example of the TI process in volleyball, which is organized and controlled by a national federation, is the Olympic Hopes Tournament (OHT). The OHT, which was first organized in 2004, exemplifies the difficulty of identifying talent in the pool of youth players. This event is organized by the Polish Volleyball Federation (PVF) for elite 14-year-old (born in the corresponding calendar year) Polish male and female players. Tournament participants represent 16 Polish voivodeships and are previously selected via regional PVF divisions. Unfortunately, players’ data from their regional PVF clubs before selection for OHTs are not available. Eight of the 12 players who qualify for the OHT from each voivodeship are obligated to meet the minimum body height requirements: 185 cm for male players and 175 cm for female players. All teams play three matches at the group stage and one or more matches in the knockout phase. The PVF sets the net height at 243 cm for boys and 223 cm for girls. During the tournament, experienced PVF coaches assess the players separately by gender and identify the players who will be offered full-time scholarships by the National Volleyball Development Program (NVDP). The final result of the tournament is the selection of male and female players for the NVDP. To the best of our knowledge, there are no reports related to the determination of the RAE or TI factors in youth volleyball tournaments similar in scale to the OHT.

Therefore, the aims of the present study were (1) to determine the differences in the quarterly age distribution between Polish youth volleyball players in the OHT and the general population, (2) to investigate the quarterly differences in anthropometric characteristics and motor test results in OHT participants and (3) to identify the criteria that determine selection for the NVDP. We hypothesized that the players selected for the NVDP would exhibit a taller body height and higher jumping ability than the unselected players would. We also hypothesized that the RAE would be most apparent in the group of males and females selected for the NVDP because of the significant role of player height in volleyball.

## Materials and Methods

### Data Collection

This study included 2,528 male (aged 14.51 ± 0.32 years) and 2,441 female (aged 14.48 ± 0.31 years) players who participated in the OHT in 2004–2015 and were selected from the official database of the PVF. The obtained data were date of birth, anthropometric characteristics, and the results of fitness tests. Data on differences in the quarterly distribution of birth dates in the Polish population (PP) were obtained from the Central Statistical Office. These data corresponded to the birth dates of the players who participated in the OHT (1989–2001). In the PP, there was no significant difference in the shape of the relative quarterly distribution of age among the studied years. All data were obtained according to the Data Protection Act in Poland, and all procedures were approved by the Research Ethics Committee of the University School of Physical Education in Wrocław.

### Procedures

To determine the quarterly birth distribution, birth-date data were listed according to the four quarters of the calendar year: Q1 (January–March), Q2 (April–June), Q3 (July–September), and Q4 (October–December). The birth dates of the male and female populations in Poland between 1989 and 2004, which correspond to the birth dates of the players participating in the OHT, were similarly arranged. The OHT competition lasted 3 days: day 1—data collection, anthropometric measurements, and motor tests; day 2—group stage and quarter-final matches; and day 3—semifinal and final matches. The day after the final OHT match, a list of players nominated for the NDVP was published.

All anthropometric and fitness data were obtained by PVF employees in preparation for performing the measurements. In the 12 tournaments from which the results were obtained, the measurements carried out by PVF employees were supervised by the same person. Before the beginning of the tests, a standardized warm-up was carried out. All measurements were taken under the same external conditions in a sports hall and at a similar time of year (October or November). For the anthropometric measurements, the players wore only shorts, and for the performance tests and jumps, they wore shorts, *t*-shirts, and volleyball-specific shoes. All testing conditions were standardized for all measurement points, including test order, hydration, and preassessment food intake.

### Anthropometric Characteristics

An electronic scale (kg) and a stadiometer (cm) were used for the anthropometric measurements. Standing reach stature was measured to the nearest centimeter using a yardstick vertical jump device (VolleySystem, Poland). Players were asked to stand with their feet flat on the ground, to extend their arms and hands and to mark their standing reach. Two measurements were made, corresponding to one- and two-arm standing reaches. The intraclass correlation coefficient for test–retest reliability and technical error of measurement (test–retest period of 1 h) in 30 youth male players was 0.24 (*p* < 0.01), which corresponded to 0.1 kg for body weight, 0.83 (*p* < 0.01), and 0.1 cm for body height and 1.18 and 1 cm for standing reach.

### Vertical Jump and Block Reach

Vertical jump height was calculated as the highest point reached during a countermovement jump with an arm swing from a standing position. Block reach was measured to the nearest centimeter, and the best value obtained from three trials of countermovement jumps with arm swings was used for the analysis for male and female players, respectively. The players were then instructed to stand on a mark and to leap as high as possible with both legs, displacing as many vanes on the yardstick as possible. All jumps were performed using a yardstick vertical jump device (VolleySystem, Poland). The intraclass correlation coefficient for test–retest reliability (test–retest period of 1 h) in 30 youth male players was 1.97 (*p* < 0.01) for vertical jump and 0.64 for block reach (*p* < 0.01). The technical error of measurement was 1 cm.

### Spike Reach and Spike Jump

The players were asked to stand with their feet flat on the ground, extend their arms and hands, and mark their standing reach. They were then instructed to take a run-up or spike approach and to leap as high as possible with both legs, displacing as many vanes on the yardstick as possible (VolleySystem, Poland). A 5-min break between jumps was applied. The best result out of two trials was recorded. The spike jump values were calculated as the difference between the heights of the jump and the standing one-arm reach. The intraclass correlation coefficient for test–retest reliability (test–retest period of 1 h) in 30 youth male players was 0.66 for spike reach (*p* < 0.01). The technical error of measurement was 1 cm.

### Zigzag Agility Test

**FIGURE 1:**
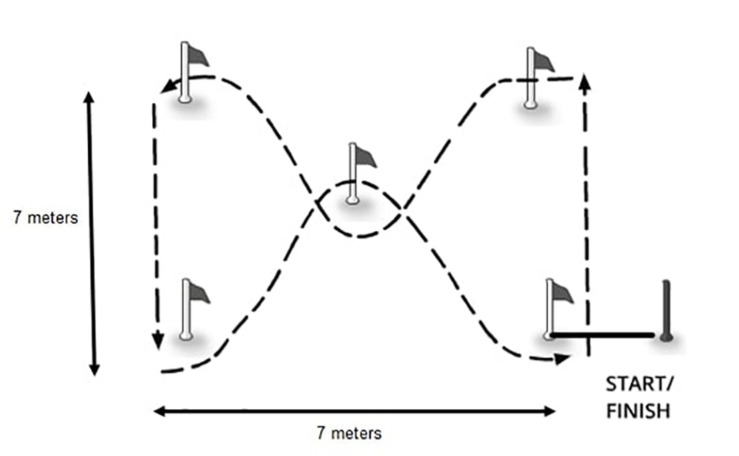
Zigzag agility test.

The zigzag agility test consisted of running at maximal speed through a 7 × 7-m zigzag course (Figure 1). Timing began with a sound signal and stopped when the subject passed through a timing gate (SECTRO Timing System, Jelenia Gora, Poland); the time was measured in hundreds of seconds. A 5-min break between trials was applied. The best result out of two trials was recorded. The intraclass correlation coefficient for test–retest reliability (test–retest period of 1 h) in 30 youth male players was 0.46 s for the zigzag agility test (*p* < 0.01). The technical error of measurement was 0.01 s.

### Statistical Analysis

Assessment of the normality of the variable distributions was performed using the Kolmogorov-Smirnov test with Lilliefors correction. Homogeneity of variance was checked, and no violations were found. The χ^2^ test was used to determine the differences between the observed and expected frequencies of a birth-date quartile. The effect size was defined by calculating Cramér’s *V*. The threshold values for *V* were set according to Cohen’s (1988) guidelines for *df* = 3, as follows: ≥0.06 (small), ≥0.16 (medium), and >0.29 (large). An independent-samples *t* test was conducted to determine the differences in anthropometric characteristics and fitness test results between selected and unselected players for each birth quarter. In addition, multivariate analysis of covariance (MANCOVA) with chronological age and age as covariates and anthropometric characteristics and motor test results as dependent variables was used to examine differences among birth quarters (independent variable). A significant α was set at 0.05. Threshold values for effect size statistics were 0.01, 0.06, and 0.14 for small, medium, and large effect sizes, respectively (Cohen, 1988). To support univariate analyses, Bonferroni *post hoc* test was used where appropriate.

Performance characteristics were analyzed using a stepwise discriminant function analysis to determine which combination of the measured characteristics optimally explained the selection of qualifying players to join the NVDP. In this analysis, the group (selected for the NVDP vs. not selected) was the dependent variable, and performance characteristics, birth quarter, and calendar age were the independent variables. The calculation included the cases for which complete data were provided. The analysis did not include the medicine ball throw because of its exclusion from the battery of tests in 2012. All calculations were performed using IBM SPSS statistical software (version 22.0, Armonk, NY, United States).

## Results

Table 1 shows the χ^2^ test results (χ^2^ = 7.9, *p* < 0.05, *V* = 0.06, a small effect for males; χ^2^ = 1.2, *p* > 0.05, *V* = 0.05, no effect for females), percentage deviations, and standardized residuals for the comparison of the OHT players and the players selected for the NVDP. The observed quarterly distributions of players selected and not selected for the NVDP were significantly different from the uniform distribution (*p* ≤ 0.001). Furthermore, an overrepresentation of young volleyball players born in Q1 and Q2 was reported for both genders. In contrast, an underrepresentation of players born in Q3 and Q4 was observed. In addition, only 6.03% of male players and 11.42% of female players selected for the NVDP were born in the last 3 months of the year. A medium effect size of the RAE was observed in each of the subsamples of volleyball players.

**TABLE 1:** Analysis of birth-date distribution by quarter of the year among Polish elite youth volleyball players.

	**Q1 (%)**	**Q2 (%)**	**Q3 (%)**	**Q4 (%)**	**Total**	**χ^2^**	***p***	***df***	***V***	**Effect**
**Players not selected for the NVDP**										
Male	818 (43.08)	532 (28.02)	328 (17.29)	220 (11.61)	1,898	285.1	<0.0001	3	0.25	Medium
Female	745 (39.98)	566 (30.39)	317 (17.01)	235 (12.62)	1,863	158.4	<0.0001	3	0.21	Medium
**Players selected for the NVDP**										
Male	135 (42.86)	91 (28.89)	70 (22.22)	19 (6.03)	315	46.5	<0.0001	3	0.27	Medium
Female	107 (37.02)	88 (30.45)	61 (21.11)	33 (11.42)	289	23.9	<0.001	3	0.19	Medium
**Polish population born in 1989–2001**										
Male	779,527 (25.54)	783,084 (25.65)	792,715 (25.97)	697,344 (22.84)	3,052,670	35,957.7	<0.0001	3	0.02	—
Female	739,516 (25.56)	736,556 (25.46)	757,265 (26.17)	659,867 (22.81)	2,893,204	3,988.4	<0.0001	3	0.03	—
**OHT players vs. players selected for the NDVP Percentage deviations and standardized residuals**										
Male	+0.03% −0.09	+1.8% +0.17	−19.5% +1.49	−38.8% −2.16	2,213	7.9	<0.05	3	0.06	Small
Female	−3.2% −0.33	—	+8.9% +0.67	−4.3% −0.26	2,152	1.2	>0.05	3	0.05	—

*χ^2^_3_, χ^2^ test value; *p*, probability value; and *V*, Cramér’s *V.**

Anthropometric characteristics and results of the zigzag agility test across the four birth quarters or calendar age for each subgroup are shown in Table 2. The MANCOVA analysis demonstrated no significant main effect for birth quarter or calendar age in all OHT female players and in male players selected for NVDP. In the group of non-selected male players, the analysis demonstrated significant differences according to the quarter of birth for body height (*F* = 0.01, *p* < 0.001), spike reach (*F* = 7.33, *p* < 0.05), and block jump (*F* = 0.02, *p* < 0.001). Significant differences within calendar age were observed for body mass (*F* = 0.53, *p* < 0.01), spike jump (*F* = 2.64, *p* < 0.05), block jump (*F* = 0.4, *p* < 0.01), and zigzag agility test results (*F* = 0.01, *p* < 0.01). In addition, Table 2 shows the differences between the selected and unselected players according to birth quarter. Significant differences were found for all anthropometric variables in both genders. The selected NVDP players were taller (all *p* values < 0.001) and heavier (values from < 0.05 to < 0.001) and jumped higher (values from < 0.05 to < 0.001) than the unselected players. Regarding the mean time obtained in the volleyball agility test, the analyzed groups did not significantly differ in each birth quarter.

**TABLE 2:** Anthropometric variables and motor test results for Polish youth volleyball players across four birth quarters.

	**Q1**	**Q2**	**Q3**	**Q4**	**Covariates**			
**Male—non-selected to NDVP**	***n* = 818**	***n* = 532**	***n* = 328**	***n* = 220**	**F (CA)**	***p***	**F (Q)**	***p***
CA	14.780.1	14.490.1	14.311	14.0.1	—	—	—	—
Body height (cm)	187.55.8^†^	187.15.7^†^	187.66^†^	186.96.2^†^	3.92	n.s.	0.01	***
Body mass (kg)	74.68.8^&^	73.88.4^†^	73.99^&^	72.58.7^#^	0.53	**	5.03	n.s.
Standing one-hand reach (cm)	246.18.1^†^	245.87.9^†^	246.18.6^†^	245.58.2^†^	36.82	n.s.	15.16	n.s.
Spike reach (cm)	315.41^†^	315.09.5^†^	315.09.8^†^	314.39.8^†^	66.12	n.s.	7.33	*
Spike jump (cm)	69.38^&^	69.27.9^&^	69.7.90^†^	68.87.5	2.64	*	1.75	n.s.
Standing two-hand reach (cm)	242.47.8^†^	242.38.1^†^	242.388.5^†^	241.98.3^#^	35.77	n.s.	4.97	n.s.
Block reach (cm)	294.58.3^†^	294.18.6^†^	293.99.4^†^	293.49.3^#^	32.83	n.s.	34.68	n.s.
Block jump (cm)	51.96.6^#^	51.66.8^†^	51.76.8	51.67.2	0.40	**	0.02	***
Volleyball Agility test (s)	14.80.8	14.90.8	14.90.8	14.90.8	0.01	**	0.15	n.s.

**Male—selected to NDVP**	***n* = 135**	***n* = 91**	***n* = 70**	***n* = 19**				

CA	14.810.1	14.510.1	14.320.1	14.10.1	—	—	—	—
Body height (cm)	193.85.1	193.34.9	193.85.6	193.26.4	0.00	n.s.	0.56	n.s.
Body mass (kg)	77.16.8	77.87.7	76.97.0	77.07.3	0.86	n.s.	1.47	n.s.
Standing one-hand reach (cm)	254.47.2	254.26.9	254.18.2	253.89.6	0.48	n.s.	1.90	n.s.
Spike reach (cm)	326.38.7	326.68.5	326.89.2	322.17.3	0.03	n.s.	2.42	n.s.
Spike jump (cm)	71.98.5	72.48	72.67.4	68.39.6	0.21	n.s.	0.16	n.s.
Standing two-hand reach (cm)	250.77.3	250.16.6	250.47.9	248.29.4	0.24	n.s.	1.29	n.s.
Block reach (cm)	304.1	303.27.3	303.57.6	300.69.6	0.16	n.s.	0.66	n.s.
Block jump (cm)	53.4	53.15.7	53.15.9	52.46.4	0.01	n.s.	0.13	n.s.
Volleyball Agility test (s)	14.7	14.90.9	14.70.8	14.90.9	1.63	n.s.	3.39	n.s.

**Female—non-selected to NDVP**	***n* = 745**	***n* = 556**	***n* = 317**	***n* = 235**	**F(CA)**	***p***	**F(Q)**	***p***

CA (years)	14.780.1	14.510.1	14.310.1	14.10.1	—	—	—	—
Body height (cm)	174.75.4^†^	174.75.6^†^	175.25.4^†^	174.55.6^†^	0.04	n.s.	0.46	n.s.
Body mass (kg)	62.57.9	62.58	62.27.7	61.58.1^#^	0.00	n.s.	0.84	n.s.
Standing one-hand reach (cm)	227.97.9^†^	227.78^†^	228.58^†^	227.38.2^†^	0.02	n.s.	0.24	n.s.
Spike reach (cm)	277.38.4^†^	277.68.6^†^	277.48^†^	276.88.9^†^	0.36	n.s.	1.05	n.s.
Spike jump (cm)	49.36.5	49.86.8^&^	49.06.1^†^	49.56.8^#^	0.90	n.s.	0.56	n.s.
Standing two-hand reach (cm)	222.225.9	222.523.7	225.77.8	223.418.4^&^	0.10	n.s.	0.32	n.s.
Block reach (cm)	260.429.2^†^	260.826.7	264.17.3	261.419^†^	0.24	n.s.	0.58	n.s.
Block jump (cm)	38.55.3	38.65.7^†^	38.44.9	38.05.2^&^	0.72	n.s.	1.40	n.s.
Volleyball Agility test (s)	16.21.1	16.11	16.21	16.31.1	0.12	n.s.	0.94	n.s.

**Female—selected to NDVP**	***n* = 107**	***n* = 88**	***n* = 61**	***n* = 33**				

CA	14.830.1	14.520.1	14.290.1	140.1	—	—	—	—
Body height (cm)	180.95.2	179.54.9	180.24.9	182.44.5	0.62	n.s.	5.13	n.s.
Body mass (kg)	65.17.8	63.36.9	62.88.6	65.37.5	0.01	n.s.	0.14	n.s.
Standing one-hand reach (cm)	235.87.5	234.37.1	235.46.9	238.39.3	0.91	n.s.	1.43	n.s.
Spike reach (cm)	288.97.6	286.97.8	287.67.1	291.08.2	0.89	n.s.	2.01	n.s.
Spike jump (cm)	53.16.9	52.66.7	52.37.4	52.77.1	0.00	n.s.	0.09	n.s.
Standing two-hand reach (cm)	229.926.8	228.628	22348.5	234.04.9	2.14	n.s.	1.31	n.s.
Block reach (cm)	270.231	269.632.8	261.456.7	275.05.2	1.88	n.s.	1.06	n.s.
Block jump (cm)	41.05.3	41.75.6	39.85.6	41.15.2	0.11	n.s.	0.23	n.s.
Volleyball Agility test (s)	16.00.9	16.11	16.11	16.20.9	0.38	n.s.	0.02	n.s.

*^#^*p* < 0.05, ^&^*p* < 0.01, and ^†^*p* < 0.001 in independent paired *t* tests.*

The stepwise discriminant analysis results are presented in Tables 3, 4. The model determined that a combination of four characteristics optimally discriminated between the players selected and not selected for the NVDP for each gender. Vertical jump (for females = 0.82, for males = 0.87), body height (for females = 0.8, for males = 0.85), and body mass (for females = 0.8, for males = 0.84) were included in both models. Spike reach (0.84) and spike jump (0.81) were the fourth variables in the male and female models, respectively. This combination of variables correctly classified 77.7% of the female players and 71.6% of the male players in terms of their selection versus non-selection for the NVDP (Table 5).

**TABLE 3:** Stepwise discriminant analysis of included variables—females.

**Step**	**Entered**	**Lambda**	**df1**	**df2**	**df3**	**Exact *F***			
						**Statistic**	***df*1**	***df*2**	***P* value**
1	Vertical jump	0.82	1	1	1,566	309	1	1,566	<0.001
2	Spike jump	0.81	2	1	1,556	162	2	1,565	<0.001
3	Body height	0.8	3	1	1,556	124	3	1,564	<0.001
4	Body mass	0.8	4	1	1,556	98	4	1,563	<0.001

*At each step, the variable that minimizes the overall Wilks’ lambda is entered. The maximum number of steps is 20; the minimum partial F for inclusion is 3.84; the maximum partial *F* for removal is 2.71; and the *F* level, tolerance, or VIN is insufficient for further computation.*

**TABLE 4:** Stepwise discriminant analysis of included variables—males.

**Step**	**Entered**	**Lambda**	***df1***	***df2***	***df3***	**Exact *F***			
						**Statistic**	**df1**	**df2**	***P* value**
1	Vertical jump	0.87	1	1	1,644	244	1	1,644	<0.001
2	Body height	0.85	2	1	1,644	141	2	1,643	<0.001
3	Spike reach	0.84	3	1	1,644	102	3	1,642	<0.001
4	Body mass	0.84	4	1	1,644	79	4	1,641	<0.001

*At each step, the variable that minimizes the overall Wilks’ lambda is entered. The maximum number of steps is 20; the minimum partial *F* for inclusion is 3.84; the maximum partial *F* for removal is 2.71; and the *F* level, tolerance, or VIN is insufficient for further computation.*

**TABLE 5:** Classification of the stepwise discriminant function analysis (*n* and %).

		**Selected for the NDVP**	**Predicted classification**		**Total**
			**Selected**	**Unselected**	
Female^a^	*n*	No	1,083	331	1,414
		Yes	37	199	236
	Correct %	No	76.6	23.4	100
		Yes	15.7	84.3	100
Male^b^	*n*	No	1,026	433	1,459
		Yes	51	208	259
	Correct %	No	70.3	29.7	100
		Yes	19.7	80.3	100

*^*a*^77.7% of the original groupings were correctly classified; ^*b*^71.8% of the original groupings were correctly classified.*

## Discussion

This study confirms the presence of an RAE in young Polish volleyball players who participate in the OHT as part of a controlled and organized TI process carried out by the national federation. As predicted, a skewed quarterly age distribution was observed in the groups selected and not selected for the NVDP. Contrary to what was hypothesized, a similar effect size of the RAE was observed regardless of whether the players were selected for the NVDP. A significant difference between the observed and expected frequencies of birth dates among the players selected for the NVDP compared to the OHT sample was observed. Additionally, the results showed that there were differences in quarterly comparisons between selected and non-selected NDVP players. Nevertheless, the multivariate analysis showed no main effects for females and selected NVDP male players. Moreover, the discriminant analysis identified the factors affecting the TI process in a group of 15-year-old volleyball players.

The identification of the RAE in Polish youth volleyball is consistent with the results of other researchers (Okazaki et al., 2011; Campos et al., 2016). However, the unexpected overrepresentation of early-born male players selected for the NVDP may be explained by gender differences in biological development and the onset of puberty (Schorer et al., 2009; Baptista et al., 2016). In 15-year-old adolescents, sex differences at puberty are significant and persist for up to 1 year in relation to age at the start of peak height velocity (Koziel and Malina, 2018). In line with this, the tests and measurements used by the PVF for the TI process seem to apply to groups of players at significantly different stages of biological development. In addition, the two-stage selection process (call-ups to voivodeship teams and selection for the NVDP after the OHT) may affect the magnitude of the RAE in Polish youth volleyball. Unfortunately, one limitation of this study is the lack of documentation regarding preselection by regional clubs and PVF coaches. On the other hand, the results of this study showed a different pattern in youth OHT participants compared to the previous studies reporting the absence of RAE in international volleyball. The equal quarter-birth distribution was reported in the highest senior level in Dutch volleyball (van Rossum, 2006) and Israeli and in female Israeli (Lidor et al., 2014) and Brazilian volleyball (Parma and Penna, 2018). Nevertheless, in a similar context, only in a research carried out by Papadopoulou et al. (2019) did the participants’ age corresponded with data obtained in this study, but that study was conducted with small samples (clubs from one city). The effect size of RAE reported in this study was equal in each group, but there was a trend of stronger discrimination against late-born male ball-game players.

The unexpected overrepresentation of early-born male players among those selected for the NVDP not only arises from physical development but also may be due to the differences in game demands between male and female volleyball. Previous studies have shown significant gender differences in volleyball game–related statistics (Joao et al., 2010; Nikolaidis et al., 2015). Men’s volleyball is characterized by a strength-based style of play, in contrast with the more technical nature of women’s games. A study by Pion et al. (2015) reported that motor coordination differentiates elite Belgian female players from sub-elite players. This argument is further supported by the results of Vargas et al. (2018), which indicated that players could achieve success in women’s volleyball even if their physical characteristics were different from those typical of male players (e.g., lower body height).

Interestingly, the differences in anthropometric characteristics and motor test results related to the quarter in which a player was born were observed only in players who were not selected to the NDVP. However, quarter-by-quarter comparisons of the mean anthropometric variables of selected and non-selected showed differences among the female players. These findings are supported by a recent study by Carvalho et al. (2020) comparing the morphological profiles of Portuguese adult female players at different levels. They suggest that “higher body mass, body height… are important for top-level performance…,” which is in line with research indicating that body height and spike jump reach are the decisive factors for the selection of junior national female volleyball players (Tsoukos et al., 2019). Conversely, previous studies have shown that anthropometric data are inefficient for discriminating between successful and unsuccessful talent-identified junior volleyball players (Gabbett et al., 2007). Note that the discriminant analysis in the present study was conducted with a decidedly larger sample.

The results of the abovementioned studies show that jumping ability, body height, and body mass are crucial for selection for the NVDP regardless of gender. This is consistent with reports showing that a high block jump characterizes the best male volleyball players (Sattler et al., 2015). However, the discriminative models presented in this study were limited and correctly classified 77.7% of female and only 71.8% of male volleyball players according to their selection or non-selection to the NVDP. This is in contrast to the handball study conducted by Mohamed et al. (2009), which reported a correct classification rate of 87.2%. Notably, that study and other previously mentioned studies obtained simple anthropometric measurements without performing a detailed body composition analysis (i.e., fat free mass), which may indicate significant errors in the predictability and efficiency of the TI process in adolescents, in whom relative body weight seems to be more important (Chung, 2015).

Some aspects of the present study need to be put into perspective. One limitation of this study is the closed settings of the zigzag agility test that was used, which may not directly respond to game-related demands of volleyball. A player who changes direction quickly and efficiently is not necessarily effective in the game, for example, in his/her reaction to a ball flying at high speed (Young, 2015). However, as in previous studies, there was no significant difference between selected and unselected players in test results based on planned change-of-direction (Gabbett et al., 2007; Tsoukos et al., 2019). Our findings support this thesis, and no significant difference in zigzag agility test results was reported between selected and non-selected players. Nevertheless, the ability to change direction efficiently may be a factor for TI in female volleyball players, but only in relation to open tasks and decisive processes (Balser et al., 2014). We suggest including open-skilled agility tests in national federation and club TI processes for youth volleyball.

It is worth highlighting that the strength of the study was the use of a representative large data sample taken from the whole country over 14 years. However, in this study, it was impossible to consider quantified assessments of the volleyball skills of the OHT players because of the lack of documentation by the PVF. Another limitation of this study is the lack of data regarding the players’ positions on the court. In this case, such a difference may be caused by the earlier discrimination of relatively later-born players who can play in youth volleyball only as defensive players. A previous study reported differences in somatotypes between setters and centers in elite adult volleyball players (Duncan et al., 2006; Giannopoulos et al., 2017). In line with this, future studies about the TI process in youth volleyball using similar sample sizes should include players’ positions on the court.

Considering the findings and limitations of this study, several practical implications can be drawn for policymakers and trainers in the context of the TI process and the RAE in youth volleyball. First, we suggest a rethinking of the TI model in youth volleyball to account for the complexity of the RAE phenomenon and gender differences. It seems unreasonable to adopt the same criteria for assessing groups at different stages of biological development. Second, national federations and clubs should attach greater importance to the consistent collection of information from the TI process. Third, open-skilled agility tests tend to have more value in identifying talented players than tests based only on change of direction.

## Conclusion

The results of these studies confirm the existence of an RAE in youth volleyball and highlight a trend in the selection of male athletes with greater body weight and height and better jumping ability than their unselected counterpoints. We suggest that TI process in youth volleyball be designed based on complexity of the RAE phenomenon and gender differences in maturity and different anthropometric and motor demands for each player’s position on the court.

## Data Availability Statement

All datasets generated for this study are included in the article/supplementary material.

## Ethics Statement

All data were obtained according to the Data Protection Act in Poland, and all procedures were approved by the Research Ethics Committee of the University School of Physical Education in Wrocław. Written informed consent from the participants’ legal guardian/next of kin was not required to participate in this study in accordance with the national legislation and the institutional requirements.

## Author Contributions

KR: Conceptualization, investigation, and writing original draft. AR: Formal analysis. KR and AR: Funding acquisition, supervision, writing – review and editing. All authors contributed to the article and approved the submitted version.

## Conflict of Interest

The authors declare that the research was conducted in the absence of any commercial or financial relationships that could be construed as a potential conflict of interest.
